# Monocarboxylate transporter functions and neuroprotective effects of valproic acid in experimental models of amyotrophic lateral sclerosis

**DOI:** 10.1186/s12929-022-00785-3

**Published:** 2022-01-10

**Authors:** Asmita Gyawali, Sana Latif, Seung-Hye Choi, Seung Jae Hyeon, Hoon Ryu, Young-Sook Kang

**Affiliations:** 1 College of Pharmacy and Drug Information Research Institute, Sookmyung Women’s University, Cheongpa-ro 47-gil 100 (Cheongpa-dong 2ga), Yongsan-gu, Seoul, 04310 Republic of Korea; 2 Brain Science Institute, Korea Institute of Science and Technology, Seoul, 02792 South Korea

**Keywords:** Amyotrophic lateral sclerosis, Monocarboxylate transporter 1, Sodium-coupled monocarboxylate transporter, Neuroprotection, Valproic acid

## Abstract

**Background:**

Amyotrophic lateral sclerosis (ALS) is a devasting neurodegenerative disorder for which no successful therapeutics are available. Valproic acid (VPA), a monocarboxylate derivative, is a known antiepileptic drug and a histone deacetylase inhibitor.

**Methods:**

To investigate whether monocarboxylate transporter 1 (MCT1) and sodium-coupled MCT1 (SMCT1) are altered in ALS cell and mouse models, a cellular uptake study, quantitative real time polymerase chain reaction and western blot parameters were used. Similarly, whether VPA provides a neuroprotective effect in the wild-type (WT; hSOD1WT) and ALS mutant-type (MT; hSOD1G93A) NSC-34 motor neuron-like cell lines was determined through the cell viability assay.

**Results:**

[^3^H]VPA uptake was dependent on time, pH, sodium and concentration, and the uptake rate was significantly lower in the MT cell line than the WT cell line. Interestingly, two VPA transport systems were expressed, and the VPA uptake was modulated by SMCT substrates/inhibitors in both cell lines. Furthermore, MCT1 and SMCT1 expression was significantly lower in motor neurons of ALS (G93A) model mice than in those of WT mice. Notably, VPA ameliorated glutamate- and hydrogen peroxide-induced neurotoxicity in both the WT and MT ALS cell lines.

**Conclusions:**

Together, the current findings demonstrate that VPA exhibits a neuroprotective effect regardless of the dysfunction of an MCT in ALS, which could help develop useful therapeutic strategies for ALS.

## Background

Amyotrophic lateral sclerosis (ALS) is a progressive neurodegenerative disease that affects both upper and lower motor neurons, which originate from the brain and spinal cord, respectively [1] The two types of ALS with respect to the cause are familial ALS and sporadic ALS; the latter is linked to mutations in several genes, such as superoxide dismutase 1 (*SOD1*), fused in sarcoma (*FUS*), TAR DNA-binding protein (*TDP*), and chromosome 9 open reading frame 72 (*C9ORF72*) [2, 3]. Among these, the *SOD1* gene mutation is the best studied because the clinical and pathological features are similar to those of human familial ALS [2, 4, 5]. ALS is linked to pathological features such as protein misfolding, impaired axonal transport, glutamate excitotoxicity, oxidative stress, endoplasmic reticulum (ER) stress, neuroinflammation, and mitochondrial dysfunction [3]. In *SOD1* mutant models, essential transporters such as monocarboxylate transporters (MCTs), large neutral amino acid transporter 1 (LAT1), and organic cation/carnitine transporters 1 and 2 (OCTN1 and 2), are reportedly altered [6–9].

MCTs belong to the solute carrier (SLC) transporter superfamily and facilitate the passive transport of monocarboxylates, such as lactate, pyruvate, butyrate, ketone bodies, and many others, via proton coupling [10, 11]. Furthermore, two sodium-coupled MCTs (SMCTs), SMCT1 (SLC5A8) and SMCT2 (SLC5A12), are highly expressed in neurons, the brain, retina, intestine, and kidney and transport energy substrates, such as butyrate, lactate, pyruvate, nicotinate, and salicylate, via proton and sodium coupling [10, 12, 13]. SMCTs and MCTs show similar substrate specificities, despite differences in their identities and driving forces [14]. MCTs are also essential for the transport of nutrients and drugs into cells for metabolic and pH regulation and to maintain homeostasis. Neurons obtain monocarboxylates from the brain via MCTs, which strongly participate in energy metabolism during the fight against neurological disorders [13, 15].

Valproic acid (VPA) is a branched short-chain fatty acid that dissociates to form valproate ions in the body, and its half-life is 9–16 h [16]. VPA is widely used as a mood-stabilizing and antiepileptic drug, particularly for children with epilepsy and convulsive seizures [17]. Neuroinflammation caused by excessive generation of reactive oxygen species, such as superoxide anion (O_2_^−^), and inducible nitric oxide synthase in neurons can be inhibited by VPA administration [18]. In addition, VPA exerts neuroprotective effects by reducing cortical neuronal cell death from ischemia, inhibiting apoptotic cell death, and reducing oxidative and ER stress-induced motor neuronal cell death by inhibiting cytochrome c release [19]. These neuroprotective actions of VPA are linked to direct inhibition of histone deacetylase (HDAC), as well as to its effects on signaling pathways, such as the upregulation of the antiapoptotic protein in B-cell lymphoma 2 [20]. Moreover, the combination therapy with VPA and lithium remarkably reduces motor neuronal cell death induced by mitochondrial disturbances and oxidative stress in in vitro cultured neurons and also improves the survival rate of SOD1 ALS model mice [16, 21]. However, the VPA transport mechanism via MCTs is still unknown in motor neurons. Therefore, it is necessary to investigate how VPA transport is regulated under ALS stress conditions. Hence, in this study, we investigated the VPA transport mechanism and therapeutic effects under pathophysiological conditions using a motor neuron-like cell model of ALS.

## Methods

### Materials

[^3^H]Valproic acid (VPA; specific activity, 20 Ci/mmol) was purchased from American Radiolabeled Chemicals Inc. (St. Louis, MO, USA). All other chemicals and reagents were purchased from Sigma-Aldrich (Merck group; St. Louis, MO, USA) and are labelled as a high-grade commercial product.

### Amyotrophic lateral sclerosis mouse model

Male transgenic ALS mice of the mSOD1 (G93A) H1 high-expresser strain (Jackson Laboratories, Bar Harbor, ME, USA) were bred with females with a similar background (B6/SJLF1). Postmortem spinal cord tissue sections were used in this study. The animal study protocol has been approved by the institutional animal care and use committee of the Korea Institute of Science and Technology (IACUC approval no. KIST-2021-05-062).

### Cell culture

Motor neuron-like (neuroblastoma × spinal cord) NSC-34 wild-type [WT, (NSC-34/hSOD1^WT^)] and mutant-type [MT, (NSC34/hSOD1^G93A^)] cell lines were cultured following previously described methods [6, 22]. These cell lines were grown in type I collagen-coated petri dishes supplemented with high-glucose Dulbecco’s modified Eagle’s medium (DMEM; Hyclone, Salt Lake City, UT, USA) added with 10% (v/v) fetal bovine serum (Hyclone, Salt Lake City, UT, USA), 100 U penicillin/mL, and 0.1 mg streptomycin/mL, incubated at 37 °C under 5% CO_2_. After reaching confluence, cell lines were cultured and seeded on type I collagen-coated 24 (1 × 10^5^) cells/well plates (BioCoat, Kennebunk, ME, USA), and were then incubated at 37 °C for 48 h.

### Cellular uptake study of [^3^H]VPA by WT and MT ALS cell lines model

The [^3^H]VPA (0.5 µCi, 125 nM/well/200 µL) cellular uptake was performed as previously described [8, 9]. An extracellular fluid (ECF) buffer was prepared and used to wash cells thrice (1 mL) at 37 °C and 4 °C [23]. The radiolabeled compound [^3^H]VPA was dissolved in the ECF buffer in the presence or absence of specific inhibitors or substrates. Then, the uptake experiment was carried out on the NSC-34 cell lines following the designated time and pH. The uptake rate [cell-to-medium ratio (µL/mg protein)] was calculated using the following formula (1):

1 $${\text{Cell}}/{\text{medium }}\,{\text{ratio}} = \frac{{\left[ {3{\text{H}}} \right]{\text{dpm}}\,{\text{ per}}\,{\text{ cell}}\,{\text{ protein}}\,\left( {{\text{mg}}} \right)}}{{\left[ {3{\text{H}}} \right]{\text{dpm}}\,{\text{ per}}\,{\text{ medium}} \,\left( {{\mu L}} \right)}}$$

To maintain the sodium-free environment in the ECF buffer, NaCl and NaHCO_3_ was replaced with chloride and N-methyl-d-glucamine (NMG), respectively [23]. This Na^+^ free buffer was created to assess the effect of ions (Na^+^) on the [^3^H]VPA transport into the NSC-34 cell lines. Similarly, to observe the effect of pH, the ECF buffer was adjusted to pH 6.0–7.4 using HCl or NaOH following a previously described experimental procedure [6].

### Estimation of kinetic parameters for [^3^H]VPA uptake in WT and MT ALS cell lines model

To check for concentration dependence, unlabeled VPA ranging from 0 to 10 mM was used in the [^3^H]VPA uptake for 10 s at pH 7.4. Then, the kinetic parameters were calculated and fitted to the Michaelis–Menten constant (K_m_) and maximum uptake rate (V_max_) through the non-linear least-squares regression analysis using Eq. (2):

2 $${\text{V}} = {\text{V}}_{\max } \cdot{\text{C}}/\left( {{\text{K}}_{{\text{m}}} + {\text{ C}}} \right) + {\text{ K}}_{{\text{d}}} \cdot{\text{C}}$$

where, V and C are the initial uptake rate of [^3^H]VPA and the concentration of the unlabeled compound, respectively, V_max_ represents the maximum uptake rate for the saturable component, and K_d_ denotes the first order constant for the non-saturable component. For the Eadie-Hofstee plot, the saturable components were plotted by subtracting the non-saturable uptake from the total uptake rate.

Similarly, half maximal inhibitory concentration (IC_50_) values were calculated by converting the net [^3^H]VPA uptake to percentage of inhibition in the presence of edaravone, ibuprofen, and salicylic acid. The obtained values were plotted using Sigma plot version 12, and were subjected to nonlinear regression (sigmoidal plot).

### Western blot analysis for MCT1 (Slc16a1) and SMCT1 (Slc5a8/AIT)

Brain tissue was homogenized in 2.5 volume of 50 mM Tris–HCl/10% glycerol/5 mM magnesium acetate/0.2 mM EDTA/0.5 mM DTT. Protein concentration was determined using a protein assay kit (Bio-Rad, Hercules, CA, USA). Then, 25 µg of protein was subjected to SDS/10% PAGE and blotted with primary antibodies (1:1,000 dilution) [MCT1 (SLC16A1) (Cat. No.: AMT-011, Alomone Labs, Israel) and SMCT1 (SLC5A8/AIT) (Cat. No.: ab99064, Abcam, Cambridge, MA, USA)]. β-Actin (ACTB) was used as a protein loading control (1:10,000 dilution) (Cat. No.: sc-47778; Santa Cruz, USA) on the same membrane.

### Histopathology and bright field microscopy

To determine MCT1 (SLC16A1) and SMCT1 (SLC5A8/AIT) immunoreactivity changes, WT littermate (n = 5) and SOD1 (G93A) mice (n = 5) at 120–150 days of age were perfused using 4% buffered paraformaldehyde in PBS. Their spinal cords were extracted and serially sectioned at 30 µm thickness using a cryostat. Lumbar spinal cord sections (two sections per animal) were used for immunostaining to detect MCT1 and SMCT1 levels. First, the tissue sections were incubated for 1.5 h in a blocking solution (0.3% Triton-X, 2% goat serum, and 2% donkey serum in 0.1 M PBS) and were further incubated with primary antibodies MCT1(SLC16A1) (1:200 dilution) (Cat. No.: AMT-011, Alomone Labs, Israel) and SMCT1 (SLC5A8/AIT). (1:200 dilution) (Cat. No.: ab99064, Abcam, Cambridge, MA, USA), BAX (Cat. No.: sc-7480, Santa Cruz Biotech., CA, USA) (1:100 dilution), and BCL2 (Cat. No.: sc-492-G, Santa Cruz Biotech., CA, USA) (1:100 dilution) in a blocking solution at 4 °C for 24 h. After washing thrice with PBS, the tissue slides were processed with a Vector ABC Kit (Vector Laboratories, Inc., Burlingame, CA, USA). The MCT1 (SLC16A1) and SMCT1 (SLC5A8/AIT) signals were developed using the DAB chromogen (Thermo Fisher Scientific, Meridian Rockford, IL, USA). Images were then analyzed using an Olympus microscope system (Olympus, Tokyo, Japan). The semi-quantification of immunoreactivity was analyzed using ImageJ. In particular, the intensity of MCT1 (SLC16A1) and SMCT1 (SLC5A8/AIT), BAX, and BCL2 signals was counted from five to 10 motor neurons per section. A total of five sections (section/case) were used for the semi-quantification.

### Small interference RNA (siRNA) transfection in WT and MT ALS cell lines model

To scrutinize the involvement of major transporters in the transport of [^3^H]VPA in NSC-34 cell lines, mMCT1 (SLC16A1), mSMCT1 (SLC5A8), and SMCT2 (SLC5A12) transporters and the non-targeting pool (control) siRNAs (GE Healthcare Dharmacon, Inc., Lafayette, CO, USA) with Lipofectamine^®^ 2000 (Invitrogen, Carlsbad, CA, USA) were selected at a concentration of 200 nM. Using the siRNAs, NSC-34 WT and MT cell lines were transfected and incubated at 37 °C. Following the transfection, [^3^H]VPA uptake was performed for 10 s under physiological pH. Western blot analysis was also done using both the NSC-34 WT and MT cell lines [9].

### Cell viability assay

This experiment assessed the neuroprotective effect of VPA under glutamate and hydrogen peroxide (H_2_O_2_)-induced neurotoxic effects using the NSC-34 cell lines. After 70–80% confluence, the NSC-34 cell lines were exposed to H_2_O_2_ (300 µM) and glutamate (2 mM) with or without VPA (2 mM) addition, and were then incubated for 24 h. Then, an MTT [3‐(4,5‐dimethyldiazol‐2‐yl)‐2,5‐diphenyltetrazoliumbromide] solution (5 mg/mL) was added and the cells were stored in the dark at 37 °C until the appearance of a purple precipitate (formazan) [9]. Then, DMSO was added and the cells were left for 15 min; afterwards, the absorbance was measured using an Infinite F200 PRO microplate reader (Tecan Trading AG, Männedorf, Switzerland) at 550 nM. Images were captured using an EVOS XL Core Cell Imaging System (Life technologies, Thermo Fisher Scientific Korea Co., Ltd.).

### Statistical analysis

All data were expressed as mean ± standard error of mean (SEM), and were analyzed using the unpaired two-tailed student’s t-test. Differences were considered statically significant at p < 0.05.

## Results

### [^3^H]VPA transport activity is decreased in the ALS cell lines model

**Fig. 1:**
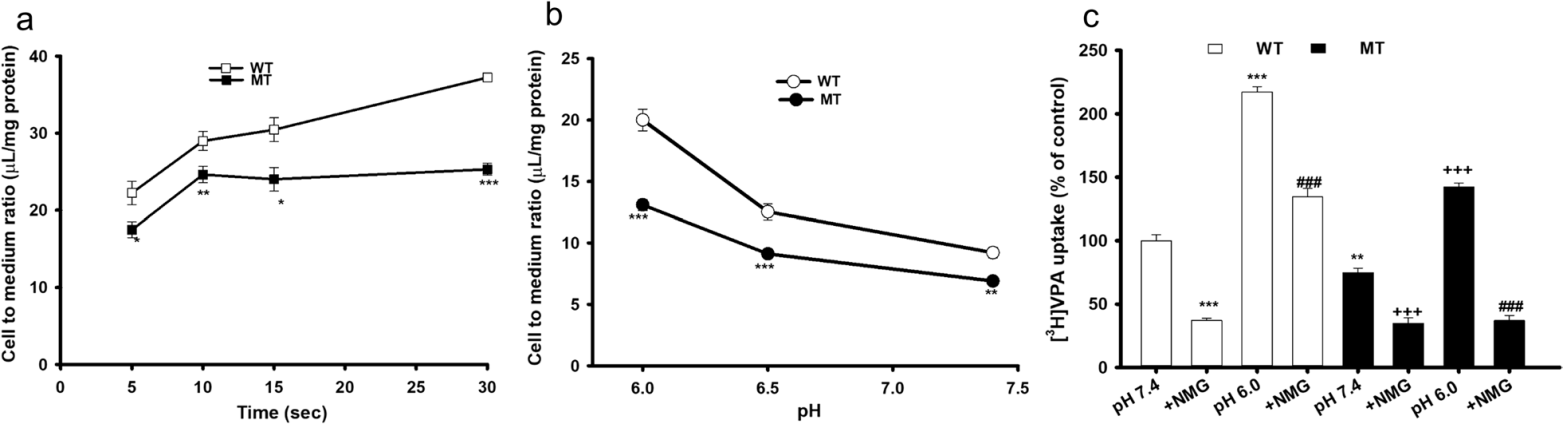
The features of [^3^H]VPA uptake is altered in the ALS cell lines model. **a** Time course of [^3^H]VPA uptake was observed for 5–30 s at pH 6.0 (**b and c**) The uptake was observed in a pH-dependent manner and sodium-free (NMG) conditions for 10 s both in WT (open space) and MT ALS (closed space) cell lines. Each value represents the mean ± SEM (n = 3–4). ***p < 0.001, **p < 0.01, *p < 0.05 versus the respective WT controls; ^###^p < 0.001 versus pH 6.0; + + + p < 0.001 versus MT pH 7.4

To examine the [^3^H]VPA transport characteristics, a time course of uptake was performed in WT and MT ALS cell lines for 5 to 30 s at an acidic pH (6.0) at 37 °C. The VPA uptake was demonstrated to be time dependent, with the transport rate rapidly and linearly increasing up to 10 s in both WT and MT ALS cell lines (Fig. 1a).

Therefore, the time was adjusted to 10 s in further VPA uptake studies. To examine the effect of extracellular pH on the VPA uptake, the pH of the ECF buffer was adjusted from acidic (6.0) to physiological (7.4). The VPA transport system was shown to be pH sensitive. The uptake rate decreased with the pH increase from acidic to physiological in both cell lines (Fig. 1b). Meanwhile, the transport rate was observed to be lower in the MT ALS cell lines than the WT cell line during the pH-dependent uptake of VPA (Fig. 1b). The transport rates were markedly lower under sodium-free conditions than in the presence of sodium during the 10-s VPA uptake by WT and MT cell lines. The uptake was pH sensitive under sodium-free conditions in both the WT and MT ALS cell lines (Fig. 1b and c). These results indicate that the [^3^H]VPA uptake is time, pH, and sodium dependent and that the transport characteristics have been altered in the ALS MT cell line compared with those in the WT cell line (Fig. 1).

### Kinetic parameters of the [^3^H]VPA uptake is altered in the ALS cell lines model

**Fig. 2:**
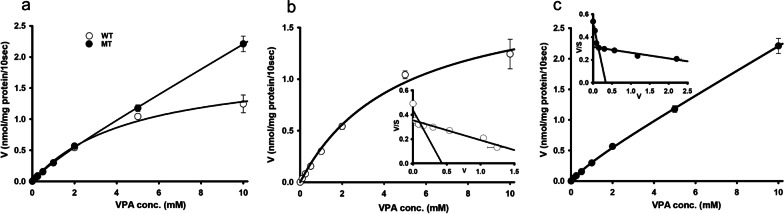
The kinetics of [^3^H]VPA uptake in WT and MT ALS cell lines model. Cold VPA at concentrations of 0–10 mM were used for 10 s of uptake under physiological pH at 37 °C (**a**) in WT (**b**) and MT (**c**) cell lines. Embedded graphs represent Eadie–Hofstee plots (**b, c**). Each point represents the mean ± standard error of the mean (*n* = 3)

Next, we clarified the reason for the alteration of the [^3^H]VPA uptake and examined the kinetic parameters of VPA transport in WT and MT ALS cell lines. The concentration dependence of the [^3^H]VPA uptake was studied for 10 s at the physiological pH in the presence of cold VPA at concentrations of 0–10 mM (Fig. 2a). The kinetic parameters determined were the Michaelis–Menten constant (*K*_m_) and the maximum transport velocity (*V*_max_) at two sites. At the high-affinity sites, the obtained *K*_m1_ values for WT and MT ALS cell line was 37.8 ± 3.0 and 19.1 ± 0.7 µM, respectively. The *V*_max1_ values for the WT and MT ALS cell lines were 0.0083 ± 0.0044 and 0.0232 ± 0.0007 nmol/mg protein/10 s, respectively. At the low-affinity sites, the *K*_m2_ and *V*_max2_ values were 4.59 ± 0.62 and 1.14 ± 0.14 mM and 1.86 ± 0.24 and 0.231 ± 0.015 nmol/mg protein/10 s for the WT and MT ALS cell lines, respectively (Table 1). The Eadie–Hofstee plots (embedded graphs in Fig. 2b and c) showed the presence of two saturable transporter systems for the transport of VPA both in the WT and MT ALS cell lines. The kinetic analysis demonstrated that the MT ALS cell lines apparently had a higher affinity and capacity for the transport of VPA at the high-affinity site and a significantly lower capacity at the low-affinity site than those of the WT cell lines (Fig. 2 and Table 1).

**Table 1:** [^3^H]VPA transporter kinetic parameters are altered between WT and MT ALS cell lines

Parameters	WT (NSC-34, hSOD1^WT^)	MT (NSC-34, hSOD1^G93A^)
K_m1_ (µM)	37.8 ± 3.0	19.1 ± 0.7**
K_m2_ (mM)	4.588 ± 0.620	1.144 ± 0.143**
V_max1_ (nmol/mg protein/10 s)	0.0083 ± 0.0044	0.0232 ± 0.0007**
V_max2_ (nmol/mg protein/10 s)	1.864 ± 0.244	0.231 ± 0.015***

Data represent the mean ± standard error of the mean. *K*_m1_, *V*_max1_ (at high-affinity sites) and *K*_m2_, *V*_max2_ (at low-affinity sites) represent the transporter affinity and maximum transport velocity, respectively. **p < 0.01 and ***p < 0.001 versus WT

### Modulation of the [^3^H]VPA uptake by monocarboxylate analogs in the ALS cell lines model

To verify whether the [^3^H]VPA cellular uptake is modulated by monocarboxylate analogs, [^3^H]VPA uptake experiments were performed with several MCT substrates and inhibitors at the acidic pH for 10 s. The presence of unlabeled valproate at concentrations of 1 and 10 mM strongly inhibited the [^3^H]VPA transport into the WT and MT ALS cell lines. Similarly, MCT substrates/inhibitors, such as acetic acid (AA), SA, 4-phenylbutyric acid (PBA), and α-cyano-4-hydroxycinnamate (CHC) at concentrations of 1 and 10 mM strongly inhibited the VPA uptake in both WT and MT ALS cell lines compared with that in the respective controls. In addition, the organic anion transporter (OAT) substrate/inhibitor *para*-aminohippuric acid (PAH) significantly reduced the VPA uptake. However, a tricarboxylate (citric acid) did not have a significant effect on the uptake of VPA in both WT and MT ALS cell lines (Table 2).

**Table 2:** Inhibitory effects of mono- and tricarboxylates on the [^3^H]VPA uptake in WT and MT ALS cell lines

Compounds	Concentration	[^3^H]VPA uptake (% of control)	
	(mM)	WT(hSOD1^WT^)	MT (hSOD1^G93A^)
Control		100 ± 4	100 ± 6
+ VPA	1	78.3 ± 4.5**	78.4 ± 1.6***
	10	35.9 ± 5.2***	37.4 ± 4.2***
+ Acetic acid	1	54.9 ± 6.7 ***	40.3 ± 5.0***
	10	24.4 ± 3.0***	15.4 ± 1.4***
+ Salicylic acid	1	64.9 ± 2.0**	78.8 ± 8.6*
	10	17.9 ± 1.5***	31.9 ± 1.6***
+ PBA	10	43.1 ± 2.2***	56.6 ± 3.0***
+ CHC	10	67.7 ± 9.0 **	74.6 ± 7.1**
+ PAH	1	71.0 ± 5.5**	58.7 ± 2.9**
	10	75.8 ± 2.3**	63.5 ± 5.5**
+ Citric acid	10	113 ± 3	125 ± 13

The VPA transport study was performed in the presence or absence of several transporter substrates and inhibitors at concentrations of 1–10 mM for 10 s at 37 °C under acidic pH (pH 6.0). The data represent mean ± SEM (n = 3–4). ***p < 0.001, **p < 0.01, and *p < 0.05 represent significant difference from the WT control. *CHC* α-cyano-4-hydroxycinnamate; *PAH* para-aminohippuric acid

Ibuprofen, a strong SMCT inhibitor, markedly inhibited (by approximately 35–65%) the VPA uptake by both the WT and MT ALS cell lines at concentrations of 1 and 10 mM. The OAT substrates edaravone (drug for ALS) and estrone 3-sulfate (E-3S) also significantly reduced the VPA uptake rate. Moreover, furosemide, a loop diuretic, and 4,4′-diisothiocyano-2,2′-stilbenedisulfonic acid (DIDS), an anion exchange inhibitor, caused noticeable depletion (approximately 40%) in VPA transport at a concentration of 1 mM both in WT and MT ALS cell lines (Table 3).

**Table 3:** Effect of drugs on [^3^H]VPA uptake in WT and MT ALS cell lines

Compounds	Concentration	[^3^H]VPA uptake (% of control)	
	(mM)	WT(hSOD1^WT^)	MT (hSOD1^G93A^)
Control		100 ± 7	100 ± 3
+ Ibuprofen	1	64.3 ± 3.6**	61.9 ± 7.5**
	10	22.3 ± 1.5***	33.5 ± 1.0***
+ Edaravone	1	80.3 ± 6.0*	81.0 ± 5.4*
	10	67.3 ± 7.6**	68.6 ± 6.3**
+ Furosemide	1	61.7 ± 6.3***	61.7 ± 2.2***
	5	77.5 ± 4.9**	82.3 ± 7.5 **
+ DIDS	1	54.8 ± 4.3***	58.8 ± 4.9***
+ Estrone − 3 sulfate	10	60.6 ± 8.4***	58.2 ± 8.5***

[^3^H]VPA uptake was analyzed for 10 s at 37 °C under acidic pH in the presence of drugs at concentrations of 1–10 mM. The data represent mean ± SEM (n = 3–4). ***p < 0.001, **p < 0.01, and *p < 0.05 represent significant difference from the respective WT and MT controls. DIDS, 4,4′-diisothiocyano-2,2′-stilbenedisulfonic acid

### Expression of MCTs in the ALS cell lines and the mouse ALS model

**Fig. 3:**
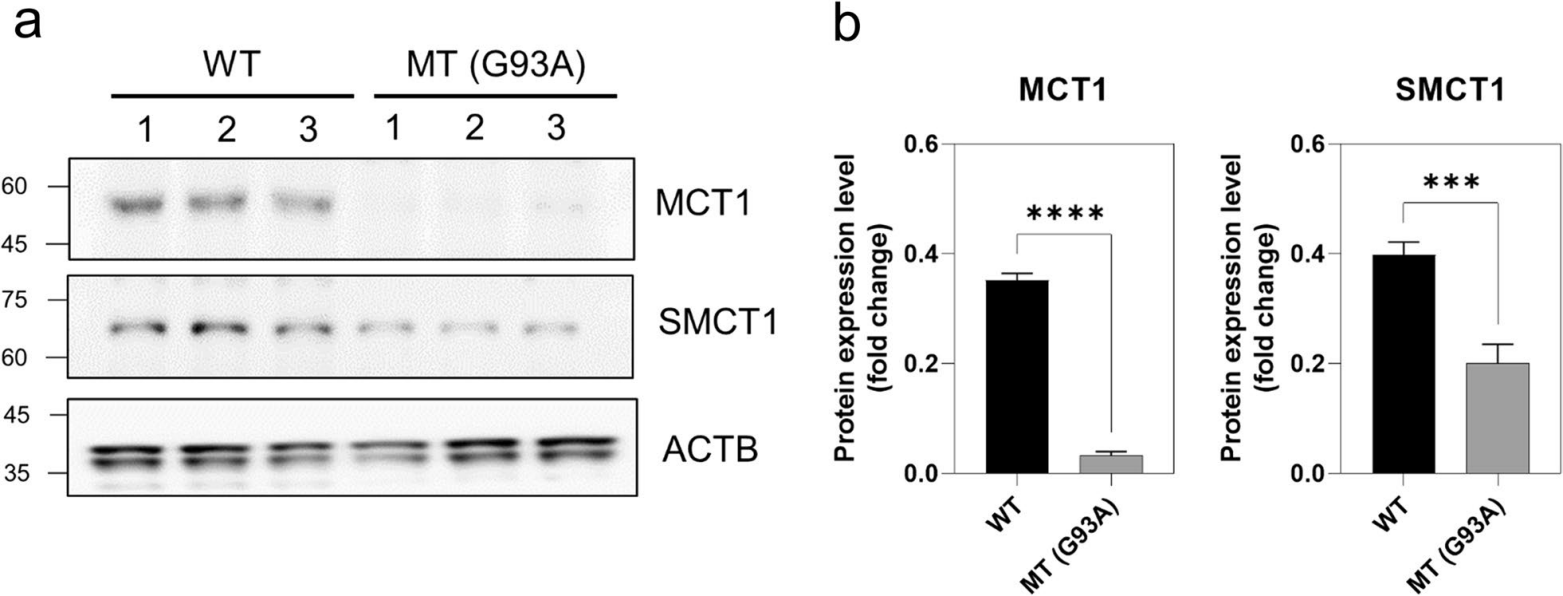
Protein expression levels of MCT1 and SMCT1 are downregulated in the ALS cell lines model. **a** Western blot analysis showed that MCT1 and SMCT1 protein levels are decreased in the MT cell line compared to the WT cell line. **b** Densitometry analysis showed that both the MCT1 and SMCT1 protein levels are significantly decreased in the MT cell line. Protein levels were normalized to those of ACTB. The bar graph data represent the mean ± standard error of the mean (*n* = 6). Significantly different at **p < 0.01; ***p < 0.001

Western blot analysis was performed to examine the protein expression level of MCT1/SLC16A1 and SMCT1/SLC5A8 in the NSC-34 (ALS model) cell lines. The protein expression pattern of MCT1 and SMCT1 were depleted in the MT cell lines than in the WT (control) cell lines (Fig. 3a). Likewise, densitometry analysis of MCT1 and SMCT1 protein demonstrated that both the MCT1 and SMCT1 proteins were significantly decreased in mSOD1-NSC-34 models compared to their respective control (WT) (Fig. 3b).

**Fig. 4:**
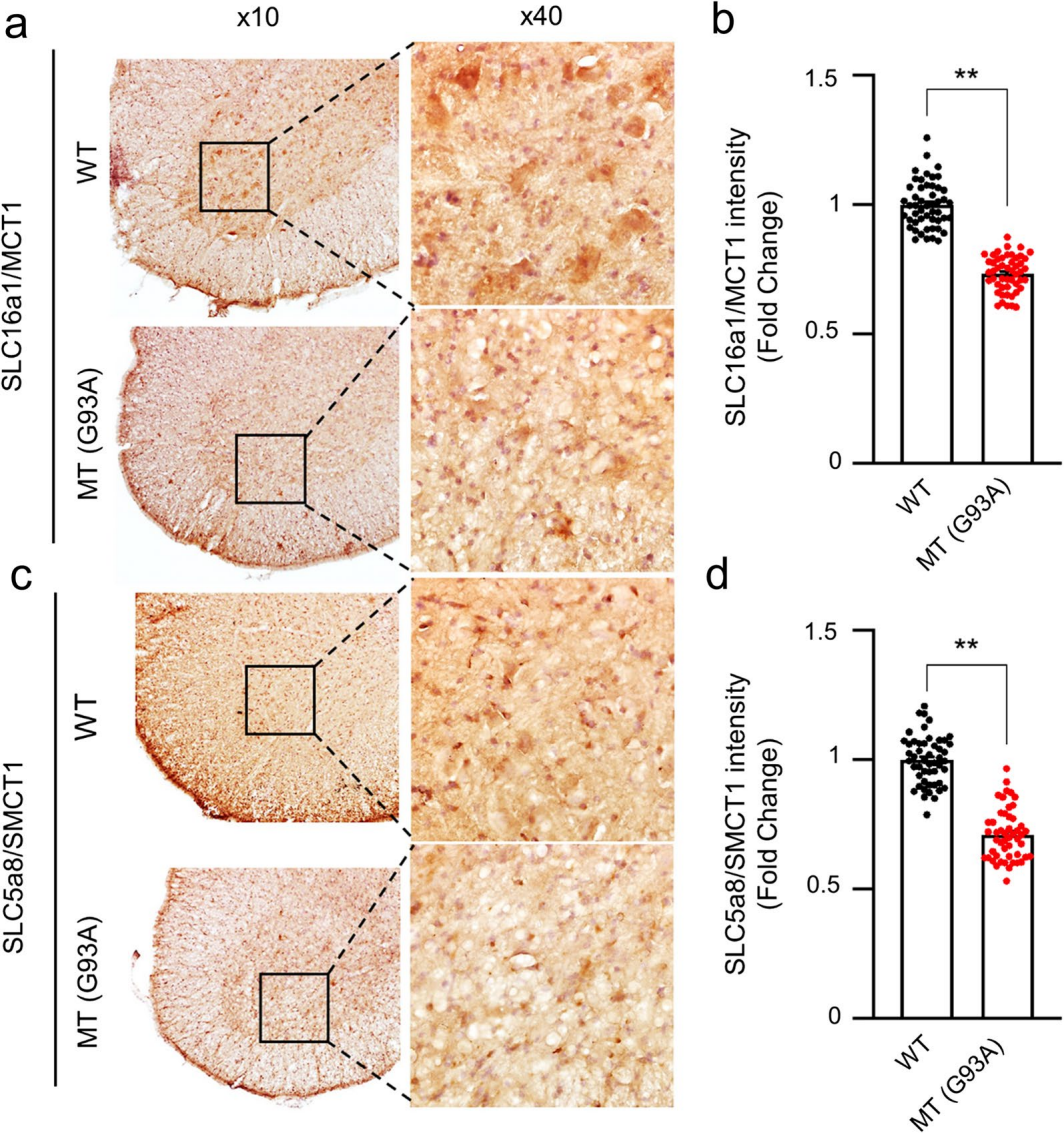
Protein expression levels of MCT1 and SMCT1 are downregulated in the spinal cord of ALS (G93A) mice. **a, b** SLC16A1 immunoreactivity was significantly decreased in motor neurons of the spinal cord of mutant SOD1 (G93A) ALS mice (n = 5) compared to WT mice (n = 5). **c, d** SLC5A8 immunoreactivity was significantly decreased in motor neurons of the spinal cord of mutant SOD1 (G93A) ALS mice (n = 5) compared to WT mice (n = 5). Cell counting, n = 50 (10 cells/case), **p < 0.001 significant difference from the WT mice

In addition, an immunohistochemistry assay was used to examine whether the protein expression of MCT1 and SMCT1 in the spinal cord was different between WT and mutant SOD1 (G93A) ALS mice (Fig. 4).

**Fig. 5:**
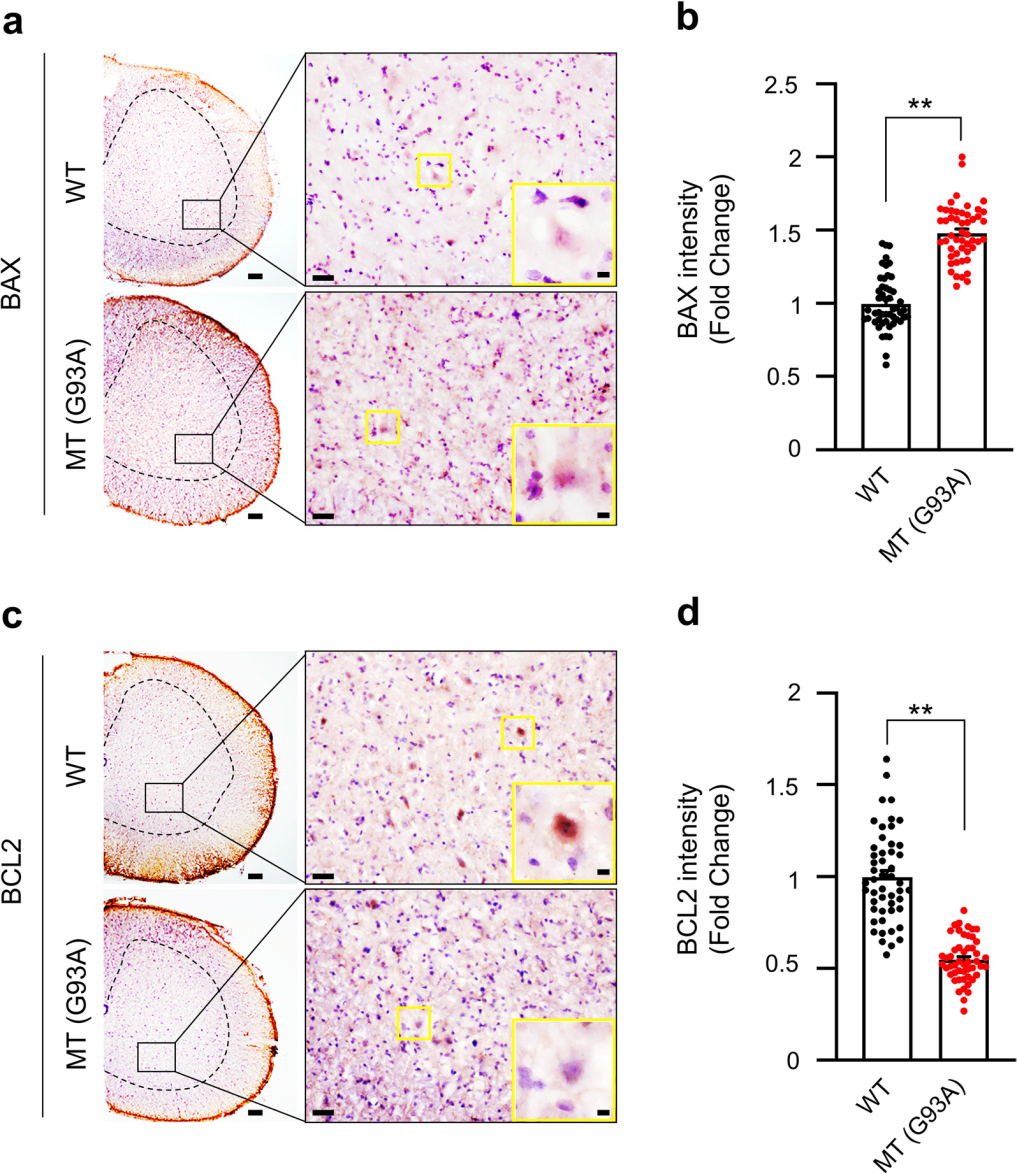
Apoptotic pathway protein expression levels are changed in the spinal cord of ALS (G93A) mice. **a, b** BAX immunoreactivity was significantly increased in motor neurons of the spinal cord of mutant SOD1 (G93A) ALS mice (n = 5) compared to WT mice (n = 5). A total of 50 motor neurons were counted [10 motor neurons × 5 section (section/case)]. **c, d** BCL2 immunoreactivity was significantly decreased in motor neurons of the spinal cord of mutant SOD1 (G93A) ALS mice (n = 5) compared to WT mice (n = 5). A total of 50 motor neurons were counted [10 motor neurons × 5 section (section/case)]. **p < 0.001 significant difference from the WT mice. Scale bars: left, 100 μm; middle, 50 μm, right (inlet) 10 μm

Immunoreactivity of MCT1 and SMCT1 was mainly observed in motor neurons, and their levels in the spinal cord gray matter were lower in ALS (G93A) mice than in WT littermate mice (Fig. 4a–d), which was similar to the western blot data in Fig. 3. These data confirm that both MCT1 and SMCT1 transporters are deleted/mutated in the MT model of ALS, resulting in low expression levels. Otherwise, in order to examine whether pro-survival or apoptotic pathway-associated protein levels are changed in the spinal cords of WT and ALS (G93A) mice, we immunostained BCL2 and BAX proteins. As expected, the immunoreactivity of BAX, an apoptosis pathway marker, was significantly upregulated in the spinal cords of ALS (G93A) mice in comparison to WT mice (Fig. 5a and b). In contrast, the immunoreactivity of BCL2, a pro-survival pathway marker, was significantly downregulated in the spinal cords of ALS (G93A) mice in comparison to WT mice (Fig. 5c and d).

### Knockdown of MCT1 and SMCT1 decreases their protein levels in the ALS cell lines model

**Fig. 6:**
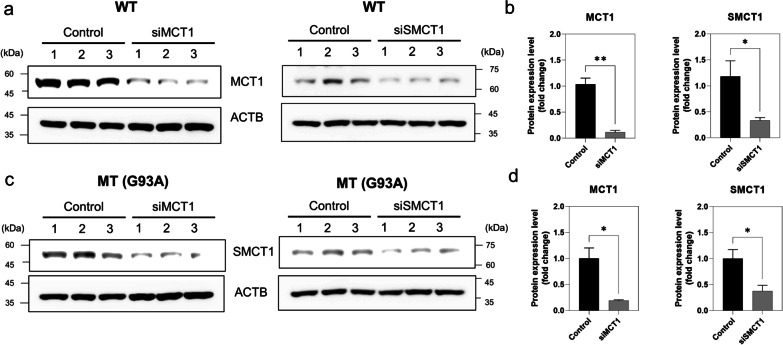
Western blot analysis of MCT1 and SMCT1 siRNAs in the ALS cell line model. Western blot analysis (**a, c**) and densitometry analysis (**b, d**) were performed in the WT (**a, b**) and MT ALS (**c, d**) cell line. Cells were transfected by siRNAs for 48 h. Protein levels were normalized to those of ACTB. The bar graph data represent the mean ± SEM (n = 3). Significantly different at *p < 0.01; **p < 0.005

Next, we examined whether *SMCT1* and *MCT1* siRNA transfection affected the protein expression levels in the NSC-34 cell lines. After 48 h of siRNA transfection, the protein expression pattern was evaluated through Western blot analysis (Fig. 6).

Among the MT cell lines, MCT1 and SMCT1 siRNA-transfected cell lines had significantly lower MCT1 and SMCT1 protein expression levels (Fig. 6c, d) than those in the WT control model (Fig. 6a, b). In addition, the protein expression levels of both transporters were depleted by more than fourfold in the MT ALS cell lines compared with those in the control (Fig. 6b, d).

**Fig. 7:**
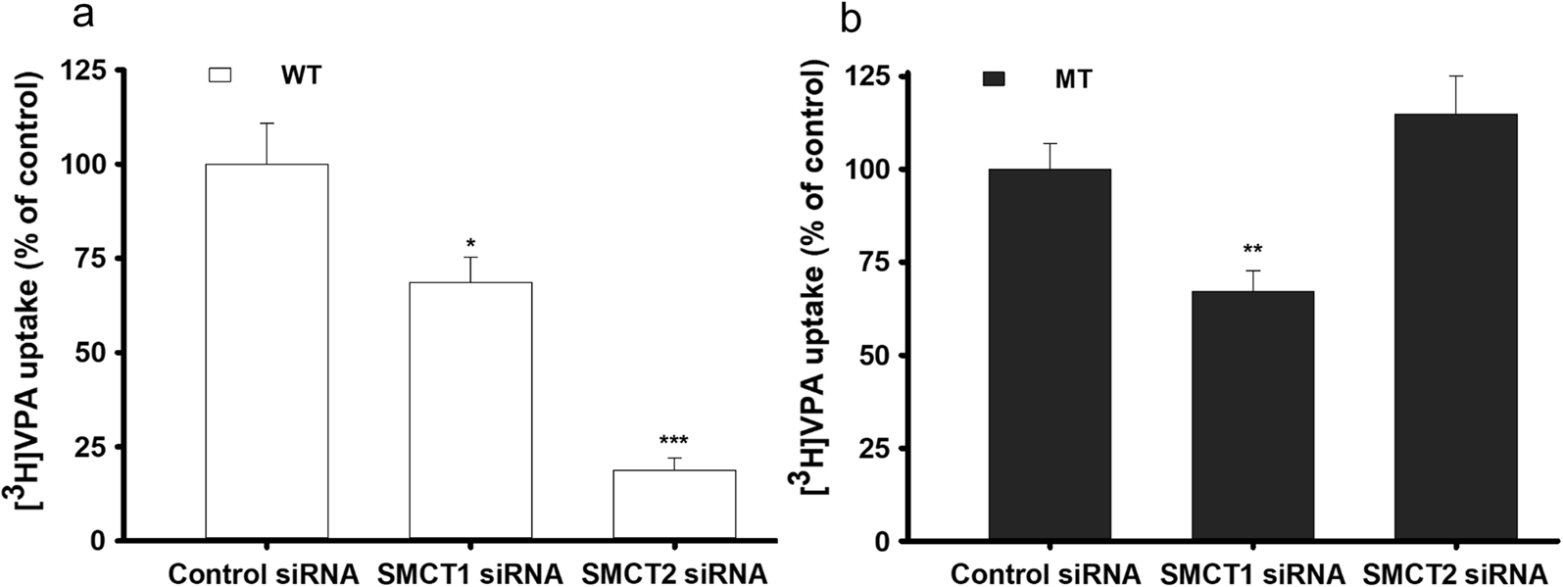
Knockdown of SMCT by siRNAs alters [^3^H]VPA uptake in the ALS cell lines model. SMCT1 and SMCT2 siRNAs significantly affects [^3^H]VPA uptake both in the WT (**a**) and MT (**b**) ALS cell line. siRNAs were transfected at a concentration of 200 nM with Lipofectamine 2000 for 24 h. [^3^H]VPA uptake was analyzed for 10 s under physiological pH. Each value represents the mean ± SEM (n = 4). ***p < 0.001, **p < 0.01 and *p < 0.05 denote significant difference from the respective controls

To examine which transporter mainly facilitated the transport of [^3^H]VPA, WT and MT cells were transfected with mouse *Smct1* (*Slc5a8*) and *Smct2* (*Slc5a12*) siRNAs. Afterward, [^3^H]VPA uptake was performed for 10 s at the physiological pH. Both SMCTs were selected because VPA is a monocarboxylate and its uptake was shown to be sodium dependent in both WT and MT cell lines (Fig. 1). *Smct1* and *Smct2* siRNA transfection significantly decreased the [^3^H]VPA uptake rate in the WT cell line (Fig. 7a), but only *SMCT1* siRNA transfection significantly decreased the uptake in the MT cell lines compared with that in cells transfected with the respective control siRNA (Fig. 7b).

Collectively, these results indicate that both SMCT1 and SMCT2 are involved in VPA influx into the WT cell lines, whereas in the MT cell lines, VPA is partly taken up by only SMCT1 (Fig. 7), which is a common transporter for VPA import into both WT and MT cell lines.

### Modulation of the [^3^H]VPA uptake by various drugs in the ALS cell lines model

**Fig. 8:**
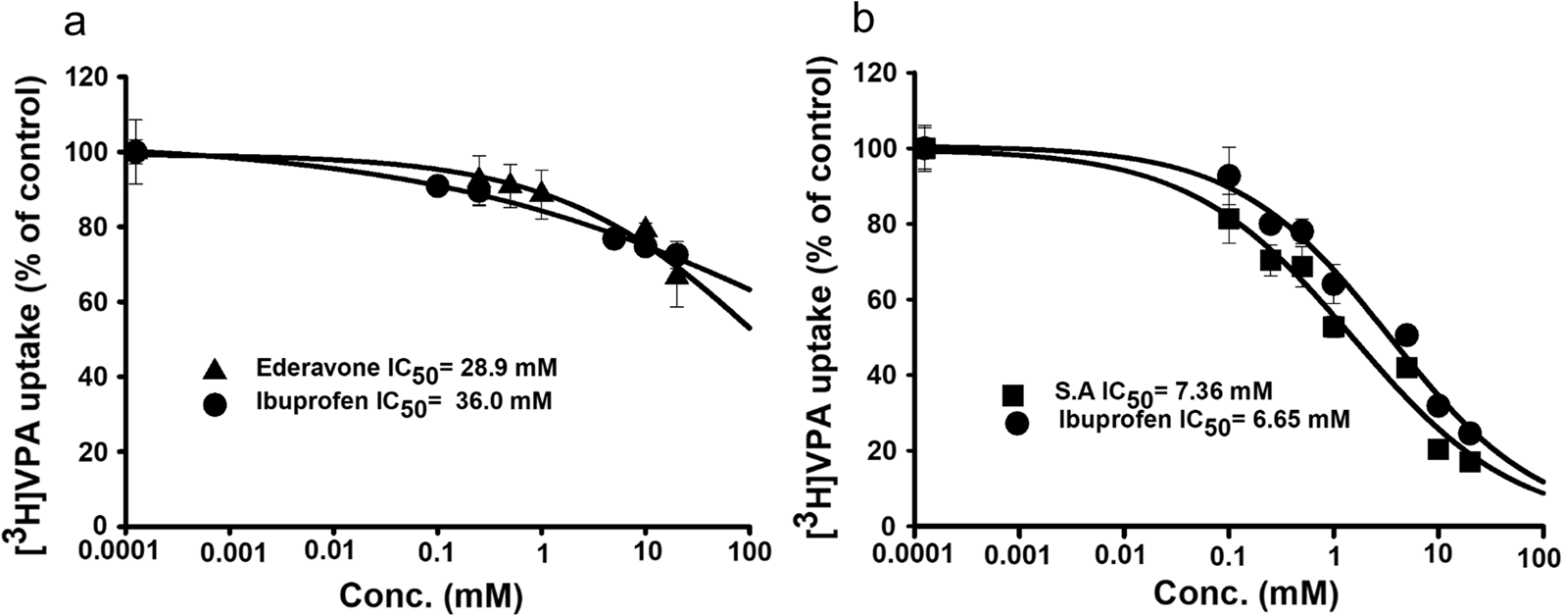
Dose–response inhibitory effects of edaravone, ibuprofen, and SA on [^3^H]VPA uptake by the MT ALS cell lines. The uptake was analyzed in the presence of edaravone, ibuprofen, and salicylate at concentrations of 0–20 mM at pH 7.4 (**a**) and 6.0 (**b**) for 10 s. The data represents mean ± SEM (n = 3)

We further tested whether various inhibitors of MCTs could modulate [^3^H]VPA uptake, which was performed using the MT cell line for 10 s at 37 °C at both pH 7.4 and 6.0. At pH 7.4, the IC_50_ values of ibuprofen and edaravone were 36.0 mM and 28.9 mM, respectively (Fig. 8a), and those of ibuprofen and SA were 6.65 mM and 7.36 mM, respectively, at the acidic pH (Fig. 8b). These results confirm that the VPA uptake is pH sensitive, as the IC_50_ values varied in a pH-sensitive manner for some of the same compounds and concentrations.

### Neuroprotective effects of VPA against glutamate- and hydrogen peroxide-induced motor neuronal cytotoxicity

**Fig. 9:**
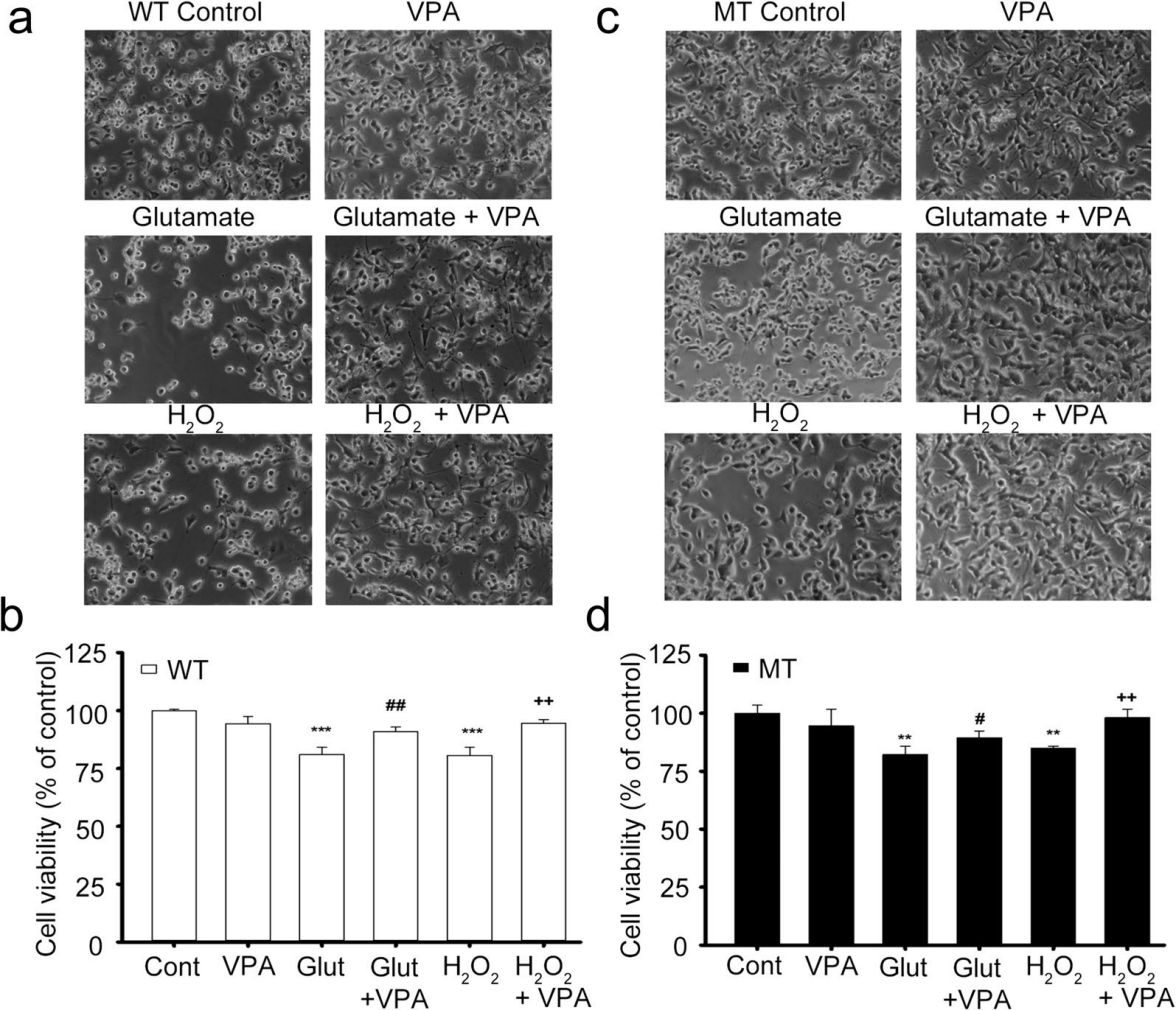
Neuroprotective effects of VPA against glutamate- and H_2_O_2_-induced neurotoxicity in ALS cell lines. VPA prevents motor neuronal cell death both in the WT (**a, b**) and MT ALS (**c, d**) cell line. Cell viability assay was observed by MTT assay after 24 h of glutamate (2 mM) and H_2_O_2_ (0.3 mM) with or without pretreatment of VPA (2 mM). Each value represents the mean ± SEM (n = 3–4). ***p < 0.001, versus control; ^###^p < 0.001, ^##^p < 0.01 versus glutamate

The neuroprotective effects of VPA against glutamate- and H_2_O_2_-induced neurotoxicity were assessed using the NSC-34 WT and MT cell lines. Preincubation with glutamate and H_2_O_2_ for 24 h significantly decreased the viability both the WT and MT cell lines (Fig. 9). However, the addition of VPA to glutamate and H_2_O_2_ restored the viability of both WT (Fig. 9a, b) and MT (Fig. 9c, d) cell line to the normal levels. These results demonstrated that VPA could have a neuroprotective effect against glutamate- and H_2_O_2_-induced neuronal cell death in both NSC-34 cell lines (Fig. 9). In addition, VPA only-treated cells did not show any significant differences in viability compared with the respective controls, indicating that 2 mM VPA is not toxic (Fig. 9).

## Discussion

VPA is a primary substrate in the mitochondrial fatty acid β-oxidation pathway [18]. It has been proposed that VPA might be beneficial for the prevention of neurodegenerative progression in ALS because its mechanism of action is similar to that of the Food and Drug Administration-approved ALS therapeutic riluzole. Both of these compounds exert an anti-excitotoxicity effect in ALS [24]. Moreover, use of the anti-excitotoxin or VPA can inhibit motor neuronal death by blocking the stimulation of glutamate receptors [25]. In the present study, the neuroprotective role of VPA was observed upon preincubation of cells with a neuroinflammatory stimulus (glutamate or H_2_O_2_).

Notably, we found, using WT and ALS MT model cell lines, that [^3^H]VPA uptake by motor neurons was time, pH, and sodium dependent (Fig. 1). The transcellular VPA transport across *human placental choriocarcinoma* (BeWo) cells and in the biomedical model *Dictyostelium* was shown to be time and pH sensitive [26, 27], similar to the data of our present study. Likewise, the transport of VPA was strongly inhibited under sodium-free conditions at both pH 6.0 and 7.4 (Fig. 1). These data indicate that H^+^ and Na^+^ symporters could be involved in the transport of VPA in the WT and MT ALS cell lines. Similarly, kinetic parameters represented a higher affinity and higher capacity transporter system expressed at the high-affinity site in the MT ALS cell line compared with that in the WT cell line (Fig. 2 and Table 1). The *K*_m_ values of VPA were in the range of 1–4 mM at the low-affinity sites in the motor neuronal cell lines (Table 1). In the previous study the potent SMCT inhibitor 5-aminosalicylic acid also exhibited the *K*_m_ value of 2.4 mM in the colonic mucosa [12]. Likewise, in earlier study the VPA transport affinity in the intestinal epithelium and brain endothelial cells was also reported to be 0.6–0.8 mM [28], which was similar to the *K*_m_ value of VPA at the high-affinity sites in the motor neuronal cell lines. In addition, the affinity and capacity of the transport system are affected by the concentrations of substrates and pH, according to previous studies. Thus, monocarboxylates transported via an SMCT showed *K*_m_ values in the range of 0.07–6.5 mM [14, 29, 30], which were similar to the *K*_m_ values of VPA in the NSC-34 cell lines.

Previous studies have shown that the inhibition rates of SMCTs and the transport mechanism of a compound depend on the tissue and structural specificity, which in turn depend on the gene expression pattern and nature of a compound. SMCT substrates such as SA, AA, and PBA block the transport function of VPA because these substrates compete for binding to the same transporter in the motor neuronal cell lines [6, 10]. The nonsteroidal anti-inflammatory drug ibuprofen acts as a blocking agent in the SMCT transport system [10, 30], thereby leading to strong inhibition of the VPA uptake in the motor neuronal cell lines. VPA is transported into rat brain microvascular endothelial cells via OAT polypeptide 2; by contrast, intestinal epithelial, brain endothelial, and choriocarcinoma placenta cells transport VPA via MCT1 in a proton-dependent manner [28, 31, 32]. In the current study, an MCT1-specific inhibitor (CHC) and OAT inhibitors/substrates (PAH, edaravone, and E-3S) also inhibited the VPA uptake both in WT and MT ALS cell lines (Table 2). VPA is transported in a tissue-specific manner and could serve as a wide-spectrum transporter inhibitor/substrate [10, 33], which explains the inhibition of the VPA transport system by an anion exchange inhibitor (DIDS), a diuretic drug (furosemide), and OAT substrates/inhibitors in the WT and MT ALS cell lines (Table 3). Although γ-hydroxybutyric acid (GHB) and 5-aminosalicylic acid are MCT substrates, these compounds follow SMCT1 transporter in rat thyroid follicular (FRTL-5) cells and colonic epithelial cells [12, 14]. SMCT substrates are known to act as HDAC inhibitors that are transported in a sodium-dependent manner and share a common substrate specificity [10]. VPA is also an HDAC inhibitor and is transported in a sodium-dependent manner. VPA uptake is inhibited by SMCT substrates/inhibitors, which further suggests that VPA follows the SMCT system in the WT and MT ALS cell lines.

A previous study has reported that MCT1, SMCT1, and SMCT2 are highly expressed in the brain and neurons, but SMCT2 tissue distribution is more restricted than that of SMCT1 [14]. In the current study, MCT1 and SMCT1 protein expression was observed in motor neurons of the spinal cord of mice. The results of western blot analysis and immunohistochemistry indicated that MCT1 and SMCT1 expression was low in the ALS MT cell line and ALS mice (Figs. 3, 4 and 5). A previous study has demonstrated that MCT1 expression is low in the spinal cord of patients with ALS and in a transgenic (SOD1^G93A^) ALS animal model, indicating that MCT1 dysfunction in oligodendrocyte MCT1 are linked to neurodegeneration [34]. The fluctuations in the levels of the transporter are probably associated with metabolic disorders and pathologies of the central or peripheral nervous system [35]. Furthermore, the knockdown of *Mct1* and *Smct1* by siRNA transfection was observed both in the WT and MT ALS cell lines resulted in an approximately fourfold reduction in the expression of MCT1 and SMCT1 compared with that in the control. Consistent with the reduced MCT levels in the ALS cell line and mouse model, the VPA uptake level was significantly lower in the MT ALS cell line than in the WT cell line (Figs. 6 and 7). Notably, glutamate or excitatory amino acid transporters, OCTN1 and 2, and LAT1 have also been linked to the downregulation of the expression of MCTs in an ALS cell line and in the transgenic (SOD1^G93A^) ALS animal model [8, 9, 36].

Because the VPA transport was found to be sodium dependent, SMCT1 and SMCT2 were selected for the identification of the major transporter involved in the WT and MT ALS cell lines. SMCT1 and SMCT2 were both found to be involved in the [^3^H]VPA transport into the WT cell lines, but SMCT1 primarily facilitated the transport into the MT ALS cell lines (Fig. 7). Overall, SMCT1 may be the common transporter involved in VPA transport in motor neuron cell lines because both SMCTs share a common substrate specificity and both transport monocarboxylates (Fig. 7). By contrast, SMCT2 is known to be a low-affinity transporter, and *Smct2* siRNA transfection significantly decreased the VPA uptake bythe WT cell line (Fig. 7). Interestingly, the VPA kinetic parameters also highlighted the presence of a lower-affinity system in the WT cell lines (Fig. 7).

High concentrations (6–36 mM) of drugs such as ibuprofen, SA, and edaravone were needed to block the biological process of the [^3^H]VPA uptake by half in the MT ALS cell line (Fig. 8) SA is a strong inhibitor, and ibuprofen is a competitive blocker of an SMCT; hence, the IC_50_ values of both compounds were in a low range, and both are competing for binding with a transportable substrate [30]. According to previous studies, 5-aminosalicylic acid exhibits a *K*_i_/IC_50_ value of 2.8 mM for inhibition of the uptake of the SMCT1 substrate nicotinate in mouse colon cell lines; however, L-lactate, ibuprofen, ketoprofen, and probenecid exhibit IC_50_ values in the micromolar range for the inhibition of the GHB uptake by FRTL-5 cells [12, 37]. It should be noted that the aforementioned IC_50_ values depended on the experimental cellular model, concentration range, and pH sensitivity.

Importantly, our current study showed that VPA treatment ameliorated glutamate- and H_2_O_2_-induced neurotoxicity in both the WT and MT ALS cell lines (Fig. 9). Consistent with our findings, a previous report has indicated that VPA has a neuroprotective role in both neuronal cell lines and mutant SOD1 ALS animal models [38]. Similar to the riluzole effect in ALS, reduces the number of apoptotic cells and prevents glutamate-induced neuronal excitotoxicity by modulating the expression and activity of *N*-methyl-D-aspartate receptors and voltage-gated sodium channels [24, 37]. According to the previous report, VPA injection protects both the in vivo rat spinal cord cells and in vitro cultured neural cells against neuronal cell death via upregulation of antiapoptotic B-cell lymphoma-extra large (Bcl-xl) expression and the downregulation of proapoptotic Bcl2 associated X (Bax) expression in the NF-κB signaling pathway which is mediated by the HDAC inhibition and decrease in autophagy [18] (Fig. 5). Also, in a transgenic mouse model of ALS, VPA supplementation can maintains normal acetylation levels which helped to prevent motor neuronal death against oxidative stress [37]. In addition, VPA acts against oxidative and ER stresses and exerts a neuroprotective effect by inhibiting protein oxidation and lipid peroxidation [37]. These mechanisms support our findings that VPA can rescue neuronal viability from H_2_O_2_- and glutamate-induced toxicity in the ALS cell line model (Fig. 9).

## Conclusions

In conclusion, the expression of MCTs (MCT1 and SMCT1) and the function of VPA transport systems were altered in ALS cellular and animal models, indicating that MCT1 and SMCT1 could be pathological markers for ALS. SMCT1 primarily facilitated the VPA transport in the ALS cell line model. VPA showed a neuroprotective role against oxidative stress and excitotoxins in the ALS cell line model. Together, VPA could be used to treat the motor neuron disease, ALS.

## Data Availability

All data generated or analysed during this study are included in this published article.

## Abbreviations

ALS: Amyotrophic lateral sclerosis
Bcl-2: B-cell lymphoma 2
*C9ORF72*: Chromosome 9 open reading frame 72
ECF: Extracellular fluid
ER: Endoplasmic reticulum
HDAC: Histone deacetylase
H_2_O_2_: Hydrogen peroxide
*K*_m_: Michaelis–Menten constant
*V*_max_: Maximum transport velocity
MCTs: Monocarboxylate transporters
mRNA: Messenger RNA
MT: Mutant type
WT: Wild type
NSC-34: Motor neuron-like (neuroblastoma × spinal cord)
NMG: N-methyl-d-glucamine
*SOD1*: Superoxide dismutase 1
SMCTs: Sodium-coupled MCTs
siRNA: Small interfering RNA
VPA: Valproic acid
